# The role of community and population ecology in applying mycorrhizal fungi for improved food security

**DOI:** 10.1038/ismej.2014.207

**Published:** 2014-10-31

**Authors:** Alia Rodriguez, Ian R Sanders

**Affiliations:** 1 Soil Microbiology, Faculty of Science, National University of Colombia, Ciudad Universitaria, Bogotá, Colombia; 2 Department of Ecology and Evolution, University of Lausanne, Lausanne, Switzerland

## Abstract

The global human population is expected to reach ∼9 billion by 2050. Feeding this many people represents a major challenge requiring global crop yield increases of up to 100%. Microbial symbionts of plants such as arbuscular mycorrhizal fungi (AMF) represent a huge, but unrealized resource for improving yields of globally important crops, especially in the tropics. We argue that the application of AMF in agriculture is too simplistic and ignores basic ecological principals. To achieve this challenge, a community and population ecology approach can contribute greatly. First, ecologists could significantly improve our understanding of the determinants of the survival of introduced AMF, the role of adaptability and intraspecific diversity of AMF and whether inoculation has a direct or indirect effect on plant production. Second, we call for extensive metagenomics as well as population genomics studies that are crucial to assess the environmental impact that introduction of non-local AMF may have on native AMF communities and populations. Finally, we plead for an ecologically sound use of AMF in efforts to increase food security at a global scale in a sustainable manner.

## Introduction

The problem of producing enough food to feed the planet, and the need for increased food security, has become all too apparent in recent years. With a global human population exceeding 7 billion, and estimates of over 9 billion by 2050, global food production will have to be greatly increased (FAO, 2006; Godfray *et al.*, 2010), especially in tropical and subtropical regions where population growth rate is increasing faster than in the rest of the world (The Future of Farming, 2011). Such yield increases exceed the current global capacity to produce food by prevalent farming practices, highlighting the need to develop new technologies and better apply long-known technologies, such as growth-promoting microbial symbionts of plants, in a more efficacious manner (Bennett *et al.*, 2013).

Poor soil fertility, particularly the availability of nitrogen and phosphorus, is the most limiting to increasing crop yields (Tilman *et al.*, 2002). Consequently, nitrogen-fixing bacteria and arbuscular mycorrhizal fungi (AMF) are the two key groups of soil microorganisms with a potential for improving nitrogen and phosphorus acquisition by crops, respectively (Cakmak, 2002; Conniff, 2013; Reid and Greene, 2013). Although significant advances are being made to get effective nitrogen-fixing bacteria to farmers in developing countries (i.e. the Bill and Melinda Gates Foundation-funded N2Africa; http://www.n2africa.org/), the application of AMF has not been well adopted or lived up to the promises, despite its enormous potential (Box 1).

In this perspective article, we consider how appropriate application of AMF could improve food security, by increasing the overall yield of important staple crops irrespective of the mechanism by which it occurs (e.g. improved phosphate acquisition, improved drought or disease resistance). By food security crops, we mean those crops that are grown because they can feed a significantly large number of people and because their yields fluctuate little during periods of major climatic perturbation. We are not intending to review here the mechanisms by which the AMF symbiosis could give a crop species more food security-enhancing properties such as increased drought resistance, disease resistance or by other systemic effects revealed by recent transcriptome profiling studies, although we recognize that these aspects are extremely important (e.g. see Salvioli and Bonfante, 2013; Zouari *et al.*, 2014). Here, we outline two major challenges to (i) effectively and (ii) safely using AMF to significantly improve production of important food security crops, especially in the tropics. We believe that both these challenges can only be resolved by the adoption of community and population ecology approaches. We propose possible ways to overcome past hurdles in applying AMF inoculation technology by respecting the general community and population ecological principles. These perspectives are based on recent ecological, community and population genetic insights, coupled with recent technological advances that allow us to better understand the ecology of these important fungi. We focus particularly on the tropics because most tropical soils are acidic and very nutrient poor, especially in bioavailable phosphate. It is in these soils where we consider that AMF could make the largest contribution, but have been most overlooked.

## Challenge 1: AMF are everywhere! A major ecological challenge to effectively using AMF in agriculture

We argue that there is a major ecological challenge to effectively using AMF to increase crop production, where agronomists cannot afford to ignore microbial community and population ecology. Thousands of published studies conducted in pots with sterile soil, where inoculated versus non-inoculated plants were compared, form the basis of the well-known potential of AMF to improve plant growth. However, all soils in which crops are cultivated already contain diverse communities of AMF and all globally important food crops naturally become colonized by AMF independently from inoculation. Consequently, expecting that introducing AMF to an already established AMF community will lead to consistent increases in crop yield is utterly simplistic. We already know that microbial community structure and diversity can greatly affect plant productivity and interactions among these microorganisms can even allow persistence of less beneficial microbes (Rosendahl, 2008; Bever *et al.*, 2013; Hart *et al.*, 2013). It is unsurprising, therefore, that there are very few published field trials with AMF that have given consistently promising results with a major food crop that is responsible for feeding a significant proportion of the global human population (perhaps with one exception; Box 2). There are additional reasons for poor adoption of mycorrhizal technology (Box 1). We propose that a better understanding of the ecology and population biology of AMF, and how introduced AMF interact with existing AMF communities and populations, is essential to developing effective AMF inocula for increasing yields of the globally important crops, namely rice, wheat, cassava, maize and potatoes.

We identify four areas where ecologists could contribute significantly to a more effective use of AMF:

- Understanding the survival and colonizing ability of introduced AMF in the presence of an existing AMF community.
- Understanding the adaptability of AMF to environmental conditions that the fungus has not previously experienced.
- The importance of within-AMF species genetic variation and how it affects plant growth.
- The need to identify whether the effect on crop yield of introduced AMF is direct or indirect, through changes to the pre-existing AMF community.

## Do introduced AMF actually establish?

**Figure 1:**
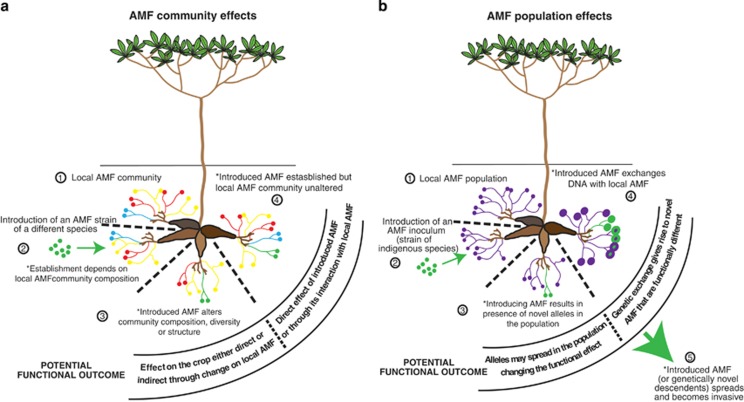
Potential effects of inoculating the tropical crop cassava with an AMF. (**a**) Effects and functional outcomes that are potentially mediated by the presence of an AMF community. (**b**) Effects and potential functional outcomes when a population of the same AMF species already exists in the field. Asterisk denotes hypotheses that can only be verified by experimental investigation as proposed in this article.

Understanding how introduced AMF interact and coexist with the local AMF community and whether this directly leads to changes in crop production is key to a successful application of AMF in agriculture. Remarkably, few field studies have directly linked yield increases with successful colonization by an introduced AMF. One field study in which yield increases were observed, with parallel increases in AMF colonization, revealed that one out of two introduced AMF inoculants may have successfully established and persisted 2 years following inoculation (Pellegrino *et al.*, 2012). The pending question is whether that particular isolate was directly responsible for the increase in biomass of the target plant or even of the higher mycorrhizal colonization observed in those roots. Nowadays, high-throughput sequencing offers a more powerful, sensitive and also quantitative technique to track the fate of the introduced AMF, both temporally and spatially and also determine whether the establishment of the AMF is influenced by the native AMF community (Figure 1a). It remains to be seen whether effective inoculation of crops with AMF will lead to significant levels of colonization by the introduced fungus, but as we explain below, this may not necessarily be important if the effects are indirect via the resident AMF community.

## Do AMF inoculants have to be preadapted to a particular soil or crop species?

Very little is known about adaptation of AMF to environmental conditions (Johnson *et al.*, 2013). A common assumption for field applications is that to be effective, the fungus has to be adapted to a given soil type or crop. Indeed, as high nutrient levels can reduce colonization by AMF, it could be more difficult for AMF to establish in more nutrient-rich soils. However, some AMF species appear to have an extremely large geographic range, suggesting a lack of specialization to certain environments (i.e. *Funneliformis mosseae* and *Rhizophagus irregularis* in Rosendahl *et al.*, 2009 and Box 3). In fact, some field studies successfully used *in vitro*-grown *R. irregularis* from arid Spanish soil in extremely nutrient-poor tropical acidic soils and with a plant (cassava) that the fungus had not previously experienced (Ceballos *et al.*, 2013).

The question of environmental adaptation in AMF has only been tangentially approached (Johnson *et al.*, 2010). Johnson *et al.* (2010) provided the first evidence, in natural ecosystems, of possible local AMF community adaptation to soil mineral nutrient levels. However, recent laboratory evidence suggests a genetic mechanism that could allow for the rapid adaptation in AMF to a change of environment (Angelard *et al.*, 2014). Strains of *R. irregularis* that had been maintained for ∼12 years on carrot roots exhibited rapid genetic change following a shift to potato and showed a very large range of phenotypic responses to a change of host. Thus, to understand whether AMF need to be adapted to a given environment, we also need to understand the mechanisms by which AMF can become adapted to a new environment.

Finally, we see little available experimental data on whether AMF that have evolved in a given environment will be more effective in that environment. Thus, we believe that in order to more effectively use AMF, it is necessary to understand the role that selective adaptation has in the effectiveness of AMF in given soils or crops.

## AMF species versus within-species diversity

AMF species richness is a major contributor to maintaining plant species diversity and ecosystem functioning (van der Heijden *et al.*, 1998a). Many field investigations have also demonstrated that crops respond differently to inoculation with different AMF species. Most of these studies demonstrating differential effects of AMF species on plants have used one individual AMF isolate as a representative of the species. However, one study shows that larger variation in plant biomass can be induced by randomly choosing two different isolates of the same species, rather than inoculating with two different species (Munkvold *et al.*, 2004). Furthermore, variation in plant growth caused by different AMF individuals from one population has been shown to have a genetic basis, highlighting genetic variation in AMF populations as a major source of variation (Koch *et al.*, 2006). Furthermore, a fivefold difference in the growth of rice was observed by inoculating with genetically different *R. irregularis* isolates (Angelard *et al.*, 2010). This calls for further studies on the functional implication of genetic diversity in AMF populations as it may be possible to breed and select more effective AMF for crop plants.

We also stress that genetic variation in crop species also needs to be considered as AMF genotype-by-crop genotype interactions may exist where crop varieties respond differently to a range of different AMF genotypes. Different plant genotypes are known to respond differently to AMF inoculation, but so far the interaction has not been investigated in sufficient detail (Hetrick *et al.*, 1995; An *et al.*, 2010).

## Direct versus indirect effects of AMF inoculation

Agricultural soils have been shown to harbour diverse AMF communities (Smith and Read, 2008). Ecological studies have clearly shown that different species of AMF induce different effects on plant growth (van der Heijden *et al.*, 1998b). Thus, it appears likely that alterations to the AMF community could potentially alter the yield of a crop without necessarily changing overall colonization levels caused by adding an additional AMF species. Introducing a non-indigenous AMF is a biotic disturbance that could alter resident AMF community structure (Figure 1a). There is no information, however, on whether AMF inoculation that results in improved crop yield is actually because of a direct effect of the introduced AMF on the plant or indirectly through a change in the local AMF community (Figure 1a). It is, therefore, highly pertinent to understand the mechanisms that govern AMF community composition in response to application of non-indigenous AMF. This is a key ecological issue in need of thorough consideration for an ecologically sustainable use of AMF (see Challenge 2). Furthermore, variable effects on crop yield of AMF application, using the same inoculum in different places, might be determined by their indirect effect on local AMF communities, which might differ in these places.

At present, there is almost no information about what determines the community composition of AMF in a given environment and, indeed, whether there are abiotic or biotic factors that allow us to predict which AMF taxa will form a given assemblage in a given locality. Without understanding of such basic ecological patterns, which we largely take for granted in plant and animal assemblages, it is impossible to even start predicting how a given AMF community might react to the introduction of an additional AMF inoculum. One way to address this is if community ecologists describe AMF community structure over a wide variety of well-described agroecosystems to identify the environmental variables that determine AMF community assemblages. With this knowledge, for a large number of sites, it would then be possible to select sites with given environments and AMF assemblages and perform experiments to observe both crop responses and AMF community responses to inoculation with one uniform inoculum. With modern molecular techniques, this very large experiment is technically possible and we see this as critical for predicting how a given AMF inoculum will affect already existing AMF assemblages.

## Challenge 2: Ecological impact of introducing non-native AMF

The second major challenge we highlight is the need to understand the ecological impact of introducing AMF into soils in which they previously did not occur. This was first addressed in a landmark publication that called for caution in global dispersal of AMF inoculants and stressed the need for environmental impact studies (Schwartz *et al.*, 2006). Recent development of new techniques means this can now be assessed and, owing to new findings, additional factors need to be taken into consideration. Without conducting the research that we highlight below, it is impossible to assess the safety and dangers associated with introducing non-native AMF. The recent developments in the analysis of microbial communities, populations and genomes make it feasible to address the following questions:

- Does an introduced AMF alter the composition and/or structure of the naturally occurring AMF community?
- Do introduced AMF persist and spread in the environment?
- If the introduced AMF species already occurs locally, are we introducing new genetic material (new alleles) into an existing AMF population?
- Will the introduced fungus undergo genetic exchange with the local AMF population and what are the functional consequences?

## Effects on AMF communities of introducing non-native AMF

An obvious question is whether the introduction of a non-native AMF significantly alters either the diversity or the composition of the existing AMF community. A number of studies have used pyrosequencing of rDNA amplicons from roots or soil, giving the first picture of AMF diversity in communities (Lindahl *et al.*, 2013; Öpik *et al.*, 2013). However, the technology needs to be suitable for large sample numbers and high replication, as well as for having the appropriate bioinformatics tools to handle very large data sets (Pagni *et al.*, 2013). At the time of writing, Illumina MiSeq technology produces ∼30 million paired-end 300 bp reads, which means that a ∼550 bp concatenated amplicon sequence should give a very large number of informative reads, making it possible to work with considerable sequence coverage per amplified sample (Smith and Peay, 2014). Pooling of barcoded amplicons from large numbers of samples into single libraries will allow large-scale studies on AMF communities from inoculated and non-inoculated soils. New more robust clustering algorithms are also being developed, such as DBC454, that are efficient for the processing of large data sets (Pagni *et al.*, 2013).

A much greater challenge is the ecological interpretation of any changes to the local AMF community. Although a decrease in AMF diversity is likely to be considered as a negative environmental impact, we still do not understand which aspects of AMF diversity (richness, evenness) favour crop growth. The experimental approach suggested above (end of Challenge 1) for investigating direct versus indirect effects should help to unravel this problem.

## Persistence and invasiveness of introduced AMF: a population genomics approach

AMF inoculants are difficult to trace in field experiments. Therefore, it has been difficult, in practice, to measure either the persistence or the invasiveness of an introduced AMF. Consequently, there is no data available on this topic (Figure 1b). A further practical problem is that the same fungal species may already be present in the native AMF community, which means that a large number of molecular markers used for tracing the fungus may already exist at the field site. However, we propose that it is potentially possible to study invasiveness and persistence of one particular AMF species, namely *R. irregularis* (Box 3), by adopting a population genomics approach, even though this fungus is already present in many agricultural fields.

With the recent publication of the reference genome of *R. irregularis* isolate DAOM 197198 (Tisserant *et al.*, 2013), it is now easier to undertake partial genome sequencing of multiple *R. irregularis* isolates and obtain a set of specific polymorphic markers to discriminate introduced *R. irregularis* strains from local ones. The technique known as random amplified polymorphic DNA sequencing is a powerful, and also an extremely reliable, high-throughput method for identifying large numbers of polymorphisms across genomes of different individuals (Baird *et al.*, 2008). Potential marker identification would then be based on polymorphisms between the introduced *R. irregularis* strain and polymorphisms of a large number of *R. irregularis* isolates whose genomes were sequenced.

## Introduction of non-native genetic material into existing AMF populations: the need for population genomics

Introducing non-native AMF at sites where the same AMF species occurs could result in the introduction of new or very different genetic material into existing fungal populations even though no new species is introduced. However, based on our knowledge of AMF genetic variation, these newly introduced individuals could potentially be genetically very different from those that are already present. This can be considered highly relevant because we already know that genetically different isolates of one AMF species can cause large differences in plant growth, even if they originate from the same population (Koch *et al.*, 2006; Angelard *et al.*, 2010).

To know the likelihood of adding new alleles into indigenous AMF populations, we believe that a detailed study of global population genomics of an AMF species is urgently needed. The fungus *R. irregularis* is an ideal candidate (Box 3). Some information on the population genetics of this fungus already exists and is intriguing. In a study of a Swiss *R. irregularis* population, where isolates originated from 1 small field (100 × 100 m^2^), genetic differences among isolates were very large (Koch *et al.*, 2004; Croll *et al.*, 2008). However, there were very few alleles in a Canadian isolate (DAOM 197198) that did not already exist in the Swiss population. This leads to the question whether *R. irregularis* populations might be locally extremely diverse, but globally not much richer. If true, it would mean that introducing inoculants of *R. irregularis* into soils from which the inoculant does not originate may pose little risk for the introduction of significant amounts of new genetic material (Figure 1b). However, only thorough population genomics studies using high-throughput polymorphism detection approaches (such as random amplified polymorphic DNA sequencing) on globally distributed *R. irregularis* isolates can help answer this question.

## Genetic exchange between introduced AMF, the existing population and its consequences

It has been demonstrated that the fungus *R. irregularis* anastomoses with genetically different individuals of the same species, giving rise to genetically novel progeny, whose effects on plant growth differ from those of the parents (Croll *et al.*, 2009; Angelard *et al.*, 2010; Colard *et al.*, 2011). Thus, anastomosis between an introduced AMF and local AMF of the same species would potentially allow non-native alleles to mix with the local population. This has the potential to change how the local AMF population affects plant growth and its competitive ability to coexist with the other species in the existing AMF community (Figure 1b).

We propose that to assess this risk, experiments are urgently needed to determine whether an introduced AMF exchanges DNA with an existing AMF population. A population genomics approach would involve: (1) genetically characterizing polymorphisms that occur in the introduced fungus, as well as in the local fungi, to develop strain-specific markers; (2) checking the absence of the markers at the introduction sites; (3) following inoculation, single spores of the fungus would have to be isolated from the field site to look for co-occurrence of markers specific to the introduced fungus and markers specific to the existing population. We consider the detection of genetic exchange in the field and its consequences to be technically challenging, but feasible with current knowledge of the *R. irregularis* genome and powerful high-throughput polymorphism detection tools.

## Conclusions

We conclude that ecological approaches at community and population scales, made possible by metagenomics and population genomics tools, can pave the way to a better informed agronomic utilization of AMF. As we point out, modern molecular techniques are already available for these studies and it is the responsibility of microbial ecologists and agronomists to take up these challenges, as their contribution could help lead to practical solutions to the problem of producing more food in a sustainable manner.

## Box 1. Enormous potential for mycorrhizal fungi to improve food security in the tropics

The potential of AMF to help increase global food security lies in the fact that all globally important food crops naturally form this symbiosis and the fungi help plants more efficiently obtain phosphate from the soil (Smith and Read, 2008). Stocks of phosphate fertilizer are rapidly being depleted (Gross, 2010). There is a simultaneous increase in demand for phosphate to help feed the growing population (Gilbert, 2009). These two combined factors represent a major threat to global food security; a threat that can potentially be reduced by better phosphate acquisition through the AM symbiosis. The potential of AMF to contribute to improved crop yields has been known for decades. However, despite an extremely strong research focus on this symbiosis,* there are remarkably few published studies demonstrating that large-scale inoculation of globally important crops, in an agricultural situation, results in significant increases in food production. The reasons for this are (taken from Ceballos *et al.*, 2013):

1. Mycorrhizal fungi are already present in all agricultural soils. Effective AMF inoculation requires yield increases over those attained in the non-inoculated crop.
2. Most AMF field trials have been conducted in temperate agroecosystems where nutrient bioavailability is high compared with most tropical soils, thus reducing the possibility of an AMF effect. In contrast, most tropical soils have low bioavailable phosphate content and high phosphate retention capacities (Friesen *et al.*, 1997). It is exactly in these soils where the application of AMF has the strongest potential to increase food production and reduce the need to apply phosphate fertilizers, but has been the most neglected.
3. AMF inoculum can only be produced with plants. Culturing AMF traditionally requires large-scale production of plants, from which the AMF inoculum can be harvested. Often this inoculum is simply soil containing AMF. It is difficult to ensure consistent inoculum quality (Gianinazzi and Vosátka, 2004), impossible to ensure that the soil/carrier is free from other microorganisms and the weight can make transport costs prohibitively high in developing countries.

*A survey of the ISI Web of Science database revealed 1441 publications on the mycorrhizal symbiosis in 2013, of which we assume at least half are probably on the arbuscular mycorrhizal symbiosis.

## Box 2. Cassava and AMF revisited

The response of most globally important crops to inoculation with AMF has been unpredictable and far from spectacular except for cassava (*Manihot esculenta* Crant*z*). Cassava is one of the most important tropical crops providing almost a billion people with a third of their daily caloric intake in 105 countries (FAO, 2010). Cassava cropping is encouraged by the FAO to improve food security because yields of cassava fluctuate much less compared with cereals during periods of climatic perturbation (FAO, 2005).

Cassava appears to be strongly dependent on mycorrhizal fungi for growth and nutrition (Howeler and Sieverding, 1983; Sieverding and Howeler, 1985). In a series of pioneering field experiments, E Sieverding and colleagues at the International Centre for Tropical Agriculture in Colombia conducted many trials inoculating cassava with AMF (their work is summarized in Sieverding, 1991). They demonstrated that large, consistent yield increases could be achieved by inoculating cassava with AMF, in the presence of an existing AMF community in Colombian soils. Also, inoculation in one crop cycle had a continued effect in subsequent crop cycles, indicating either a persistence of the introduced AMF or a beneficial durable change to the existing AMF community.

The use of AMF in cassava cropping has not been adopted because without a mass inoculum production system, the application was impractical. Currently, *in vitro*-grown *R. irregularis* can be mass produced in a sterile carrier—ensuring inoculum quality and an easy to transport product. Recently, we have successfully used *in vitro*-produced *R. irregularis* to significantly increase cassava yields in Colombia (Ceballos *et al.*, 2013). The application of the fungi requires no skill and it significantly increased cassava yields by ∼20% (with a maximal yield of 45 t ha^−1^). The effects were observed in two sites where the soils, climate and cassava variety were different.

We believe that because high-quality mass-produced and easily transportable AMF inoculum is available, the largely forgotten work, pioneered by Sieverding and Howeler (1985), needs to be revisited as it offers an important and unique opportunity where the food production and security of a globally important crop could be significantly increased through the application of AMF.

## Box 3. The case of Rhizophagus irregularis as a commercial inoculant

The AMF species *Rhizophagus irregularis* has become the model AMF species because it is easily cultured in an *in vitro* system (Bécard and Fortin, 1988) and its genome has recently been sequenced (Tisserant *et al.*, 2013). *In vitro* cultivation makes it possible to obtain fungal DNA that is free of DNA of other organisms (Koch *et al.*, 2004). The fungus has a worldwide distribution. An unpublished survey revealed that ∼300 different isolates of this fungal species probably exist in laboratories worldwide (R Savary, personal communication). This means that it is ideal for studies of AMF population genetics (Koch *et al.*, 2004; Börstler *et al.*, 2008; Croll *et al.*, 2009).

A big advantage of *R. irregularis* is that it can readily be put into *in vitro* culture and be efficiently mass produced (Bécard and Fortin, 1988). We consider this fungus to be of utmost importance in the future applications of AMF to improve food security because of: (1) its worldwide distribution; (2) its high genetic variability and variation in effects on plant growth; (3) the ability to carry out population genomics studies and marker development on this fungus; and (4) the ability to mass produce this fungus efficiently and its effects on yields of a globally important crop.

Critics may argue that it would be unwise to promote the use of one AMF species. However, at present, we see this as the most pragmatic option because of the ease to culture this fungus *in vitro* (unlike most other AMF species). Nevertheless, we emphasize that the appropriateness of using one species is entirely dependent on the niche range of the fungus, the amount of global genetic variability in this species and how much this variability contributes to variation in plant growth, all of which can only be known by undertaking the population genetics studies proposed here. However, it should be noted that for the last 8 millennia, the global human population has received the majority of its nourishment and daily calories from a only small handful of crop species and low species number has not been a major problem limiting food production, so this may be the case for AMF species as well.
